# Effect of Periodontal Disease on Alzheimer’s Disease: A Systematic Review

**DOI:** 10.7759/cureus.46311

**Published:** 2023-10-01

**Authors:** Amal Bouziane, Sara Lattaf, Lamiaa Abdallaoui Maan

**Affiliations:** 1 Department of Periodontology, Faculty of Dental Medicine, Mohammed V University in Rabat, Rabat, MAR; 2 Laboratory of Biostatistics, Epidemiology, and Clinical Research, Mohammed V University in Rabat, Rabat, MAR

**Keywords:** alzheimer’s disease, periodontal disease, review, periodontitis, dementia, oral health, inflammation

## Abstract

The aim of this review was to evaluate the relationship between periodontal disease (PD) and the onset and progression of Alzheimer's disease (AD) and to determine whether patients with PD would be at greater risk of developing AD compared to periodontally healthy subjects.

This systematic review was conducted in accordance with the Preferred Reporting Items for Systematic Reviews and Meta-Analyses (PRISMA) guidelines. An electronic search for cross-sectional, cohort, or case-control studies was conducted on five databases (PubMed, ScienceDirect, EBSCO, Web of Science, and Scopus). No restrictions were applied to the language and year of publication. Exposure was PD, and the outcome of interest was the onset and/or progression of AD. The risk of bias of the included studies was assessed using the Newcastle-Ottawa Scale (NOS) designed for non-randomized studies.

Six studies fulfilling the selection criteria were included in this systematic review. Four of the studies were of cohort design and two were of case-control design. All except one showed a significant association between PD and the risk of AD onset and progression. According to the NOS bias risk assessment, three studies were found to be of good quality, and three other cohort studies were of low quality.

Data from this systematic review indicate that patients with PD present a significantly higher risk of AD compared to individuals with healthy periodontium. However, results should be interpreted with caution given the methodological limitations found. For future research, powerful and comparable epidemiological studies are needed to evaluate the relationship between PD and AD.

## Introduction and background

Periodontal disease (PD) is an inflammatory oral disease that affects the supporting structures of the teeth (periodontium), including gingiva, periodontal ligament, cementum, and alveolar bone [1]. The disease results from complex interactions between the dental biofilm and host defense mechanisms. Bacteria and their components like lipopolysaccharides present in the biofilm induce an intensified host inflammatory response. This cascade of inflammatory response damages periodontal structures and leads ultimately to bone loss [1]. Being responsible for disability, speech impairment, low self-esteem, and reduced quality of life, PD has become a major public health concern that burdens the global healthcare system [2].

Alzheimer's disease (AD) is a progressive neurodegenerative disorder with a prevalence increasing exponentially with age [3,4]. It is characterized by an irreversible degeneration of neurons and neural connections beginning in the hippocampus and extending to the rest of the brain. People affected by Alzheimer's gradually lose cognitive abilities and autonomy. These symptoms consequently lead to advanced dementia and eventually to death. The cognitive decline that leads to AD has been related to two cardinal neuropathological features, beta-amyloid plaques (Aβ) and neurofibrillary tangles [5,6]. The amyloid plaques consist of deteriorating neuronal processes or neuritis, surrounding deposits of a central core protein called amyloid beta (or beta-amyloid). This protein is derived from a larger molecule called amyloid precursor protein, which is a normal component of nerve cells. The neurofibrillary tangle consists of abnormal accumulations of phosphorylated protein, called tau located within nerve cells. This protein is normally present in neurons. Abnormal chemical changes cause tau molecules to form tangles inside neurons.

There is a growing body of evidence in the literature suggesting a potential association between PDs and systemic diseases, particularly atherosclerotic vascular disease, pulmonary disease, diabetes, pregnancy-related complications, osteoporosis, and kidney disease as well as AD [2]. The periodontal medicine concept has been proposed to study the interrelationship between oral and systemic health. The presence of periodontal pathogens and their metabolic by-products may contribute to the body’s overall inflammatory burden, thus promoting the development of systemic conditions [2,7,8]. It has been suggested that periodontal pathogens could promote initiation or exacerbation of systemic diseases either by entering the bloodstream (bacteremia) or by stimulating an immuno-inflammatory response through the systemic release of toxins and local inflammatory mediators such as cytokines, prostaglandins, and serum antibodies into the bloodstream [2,7,8].

Most studies have indicated that Alzheimer's patients suffer from impaired oral health, a high incidence of PD, and an affected quality of life. These oral manifestations have been justified by the cognitive and motor deficits related to AD, compromising dental care and the maintenance of proper dental hygiene [9-18].

Authors have also suggested that PD may be a risk factor for AD. Hypotheses are mainly based on the involvement of periodontal pathogens and their virulence factors in the pathogenesis and progression of Alzheimer's, either by direct invasion of brain tissue or by indirect action of the bacterial load and pro-inflammatory mediators in systemic circulation [19-22]. Thus, following the elevation of the systemic inflammatory response, periodontal infection would contribute to cerebral and vascular pathologies by altering brain function and, as a result, worsen the neurodegenerative process characterizing AD [19-29].

The purpose of our systematic review was to evaluate how is PD related to the onset and progression of AD and to determine whether patients with PD would be at greater risk of developing AD compared to periodontally healthy subjects.

## Review

Methods

This systematic review was conducted in accordance with the Preferred Reporting Items for Systematic Reviews and Meta-Analyses (PRISMA) guidelines designed to report systematic reviews and meta-analyses [30,31]. The global review protocol was registered under the registration number INPLASY202080033.

Our PICOS question was formulated as follows: P (patients): adults over 40 years old with AD and without AD; I (indicator): PD; C (comparator): absence of PD; O (outcome): onset or progression of AD; S (study design): observational studies.

Search Strategy

Five electronic databases were systematically searched: PubMed/Medline, ScienceDirect, EBSCO, Web of Science, and Scopus. The following search equation was used: ("periodontal disease" OR "chronic periodontitis" OR periodont* OR periodontitis [Mesh]) AND ("Alzheimer's disease" OR dementia OR "cognitive decline" OR "cognitive impairment" OR "cognitive dysfunction" OR "Alzheimer's disease" [Mesh]). No restrictions were applied to the language and year of publication. Some limited changes were made to the search strategy due to technical restrictions in ScienceDirect, Web of Science, EBSCO, and Scopus.

Eligibility Criteria

To be included in this systematic review, studies had to be observational of cross-sectional, cohort (retrospective or prospective), or case-control design. We included studies that explored whether periodontitis was associated with AD. Exposure was PD, and the outcome of interest was the onset and/or progression of AD.

We excluded interventional studies that assessed the effect of periodontal treatment on AD; studies that examined mild cognitive impairment, cognitive impairment, cognitive decline, and dementia without mentioning explicitly AD; studies that evaluated other types of dementia; studies that assessed the impact of dementia and AD on oral health; studies that addressed the number of lost teeth without reference to AD; and studies that analyzed levels of periodontal inflammatory markers as the exposure of interest instead of PD. Experimental studies, case series, letters, comments, editorials, communications, and reviews were also excluded.

Data Extraction and Collection

Two reviewers (S.L. and A.B.) independently extracted information from each article on the first author and year of publication, country of origin, study design, sample size, age, number of years of follow-up, methods used to assess periodontitis and Alzheimer's, covariates, and results. All the selected studies were independently read by the reviewers. Every phase of data extraction was thoroughly discussed, and disagreements between researchers were resolved by consensus in consultation with a third author (L.A.).

Quality Assessment of Studies

The methodological quality of the included studies was assessed using the Newcastle-Ottawa Scale (NOS) by the two authors (S.L. and A.B.) individually. This scale has been developed to evaluate the quality of non-randomized studies [32].

The qualitative evaluation criteria comprised eight items belonging to three broad domains, namely, (1) sample selection of study groups, (2) comparability of groups, and (3) the ascertainment of either the exposure or outcome of interest for case-control or cohort studies, respectively [33]. A series of options are provided for each item, and a "star system" allows a semi-quantitative assessment of the study according to the three main domains already cited. Studies of the highest quality receive a maximum of one star for each item, with the exception of the one related to comparability that allows the assignment of two stars.

A study of good quality requires three or four stars in the selection domain, one or two stars in the comparability domain, and two or three stars in the outcome/exposure domain. A study of fair quality requires two stars in the selection domain, one or two stars in the comparability domain, and/or two or three stars in the outcome/exposure domain. Finally, a study of low quality requires zero or one star in the selection domain, zero stars in the comparability domain, and/or zero or one star in the outcome/exposure domain. Therefore, the NOS assigns up to a maximum of nine points for the least risk of bias in the three domains. Studies with less than five points are identified as representing a high risk of bias. Discrepancies in scores were resolved through discussion by the authors.

Results

Selection of Articles

The initial electronic search on all databases provided 5400 citations. After examining the titles, 4474 irrelevant articles were excluded owing to the following reasons: duplication, animal studies, reviews, letters, editorials, communications, studies in which the data did not answer the research question, and articles reporting the effect of dementia and Alzheimer's on oral and periodontal health.

The abstracts of the 926 articles selected for potential eligibility were then read and examined. Thus, 910 non-eligible articles were eliminated. They include articles assessing the oral health status of Alzheimer's patients and dementia, articles studying cognitive impairment, cognitive decline, and mild cognitive impairment without reference to AD or dementia, studies addressing the effect of tooth loss or loss of posterior dental occlusion on cognitive impairment, and articles that did not comply with our inclusion criteria.

The full text of the 16 remaining articles was read and evaluated according to the predefined eligibility criteria. Ten studies were subsequently excluded for the following reasons: exposure of interest was evaluation of periodontal antibody levels (n = 1) [34] and of plasma tumor necrosis factor-alpha (TNF-α) and antibodies to periodontal bacteria in AD and cognitively normal patients without assessment of PD (n = 1) [35]; the aim of the study was to investigate the prevalence of oral infections and blood cytokine levels in patients with AD, mild cognitive impairment, and elders without dementia (n = 1) [19] and the determination of serum antibodies to PD bacteria in participants with AD compared to antibody levels in control subjects (n = 1) [36]; verification of the presence of periodontal pathogens and pathogen-specific antibodies was made between patients with AD and subjects having other types of dementia instead of cognitively healthy patients (n = 1) [37]; the primary outcome was serum levels of inflammatory biomarkers in AD [38] and levels of expression of AD-related genes in gingival tissues without addressing the research question (n = 2) [39]; the study compared the risk of dementia in individuals undergoing various types of periodontal treatments during the follow-up period (n = 2) [40,41].

**Figure 1:**
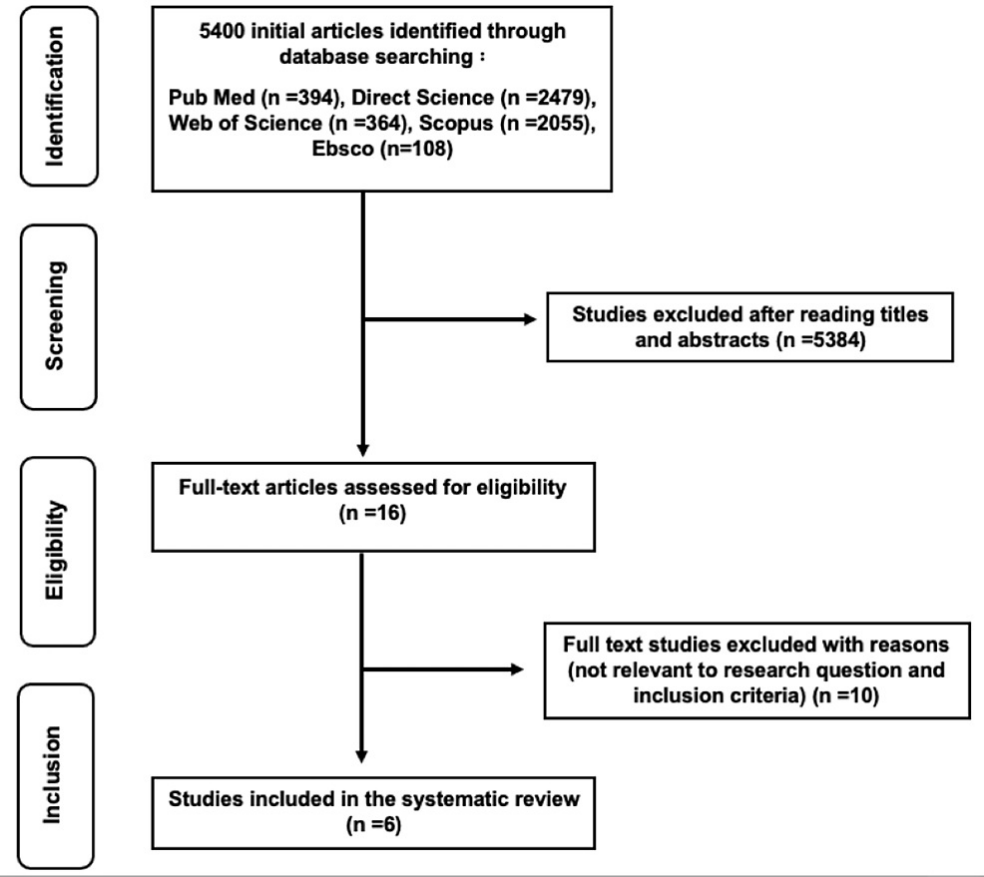
PRISMA flow diagram of search processes and results PRISMA: Preferred Reporting Items for Systematic Reviews and Meta-Analyses.

As a result, six articles fulfilling the inclusion criteria were finally included in this systematic review. The selection process is reported in the flow diagram according to the PRISMA guidelines (Figure 1).

Characteristics of the Included Studies

The characteristics of the six included studies are presented and summarized in chronological order in Table 1. They were conducted in five different countries, including France (n = 1) [42], Spain (n = 1) [43], the United Kingdom (n = 1) [44], Taiwan (n = 2) [45,46], and Sweden (n = 1) [47], and were published in English between 2012 and 2018. Four of the studies were of cohort design [42,44-46], and two were of case-control design [43,47]. Five studies identified the association between PD and the risk of developing cognitive impairment and/or various subtypes of dementia, including AD [42,43,45-47]. One study examined whether periodontitis would be associated with an increased rate of cognitive decline, regardless of the severity of dementia [44]. Arrivé et al.'s study demonstrated that PD, assessed with community periodontal index (CPI), was not associated with the risk of dementia [42]. Indeed, Gil-Montoya et al. showed that patients with severe periodontitis had almost a three-fold risk of developing dementia in comparison with subjects without periodontitis or those having mild periodontitis [43]. This association was more significant with clinical attachment loss in all patients with dementia regardless of severity. Furthermore, Ide et al. reported that during a six-month follow-up, the presence of periodontitis was linked to an increase in cognitive decline in AD patients independently of the basic cognitive state [44]. In agreement with this, Tzeng et al. found that patients with chronic periodontitis and gingivitis were 2.5 times more likely to develop dementia [45]. Similarly, Chen et al. showed an association between chronic periodontitis and AD, which became significant after 10 years of exposure [46]. Finally, Holmer et al. found that patients with AD had more marginal alveolar bone loss compared to cognitively healthy controls [47].

**Table 1:** Main characteristics of the included studies IQR = interquartile range; CPI = community periodontal index; DSM-III R = Diagnostic and Statistical Manual of Mental Disorders Revised; NINCDS-ADRDA = National Institute of Neurological and Communicative Disorders and Stroke-Alzheimer’s Disease and Related Disorders Association; HR = hazard ratio; SD = standard deviation; PI = plaque index; BI = bleeding index; CAL= clinical attachment loss; PPD = probing pocket depth; s.e. = standard error of the mean; NINDS-ADRDA = National Institute of Neurological and Communicative Diseases and Stroke/Alzheimer's Disease and Related Disorders Association; OR = odds ratio; CI = confidence interval; CDC/AAP = Centers for Disease Control and Prevention/American Academy of Periodontology; BoP = bleeding on pocket probing; ADAS-COG = Alzheimer’s Disease Assessment Scale for Cognition; sMMSE = severe Mini-Mental State Examination; ICD-9-CM = International Classification of Diseases, Ninth Revision, Clinical Modification; DSM-IV = Diagnostic and Statistical Manual of Mental Disorders IV; CP = chronic periodontitis; MCI = mild cognitive impairment; SCD = subjective cognitive decline; MMSE = Mini-Mental State Examination; MoCA = Montreal Cognitive Assessment; CDT = clock drawing test; MRI = magnetic resonance imaging; CT = computed tomography; EEG = electroencephalogram; CSF = cerebrospinal fluid; ICD = International Classification of Diseases; NIA-AA = National Institute on Aging and Alzheimer's Association; MABL = marginal alveolar bone loss.

Author/year of publication	Country	Study type	Sample size	Age	Duration of the follow-up	Periodontal evaluation	Alzheimer's diagnostic	Covariates	Results
Arrivé et al. (2012) [42]	France	Prospective cohort	405 (348 included in the analysis)	66-80 years. Median age at baseline: 70 years (IQR: 68-75)	Not clear	CPI: evaluation of gingival bleeding, calculus, and periodontal pockets	DSM-III R, NINCDS-ADRDA	Sex, education level, living condition, tobacco and alcohol intake, health status	Estimation of influence of healthy periodontal status on the risk of dementia, compared to the presence of periodontal pockets ≥ 4 mm; HR = 0.85 (p = 0.135)
Gil-Montoya et al. (2015) [43]	Spain	Case-control	409. G1 (cases): N = 180. Mild cognitive impairment (without dementia): 21; moderate to severe dementia: 123; severe dementia: 36 (70% of dementia cases had AD). G2 (controls): N = 229	>50 years. G1: 77.0 ± 7.8 (mean ± SD). G2: 78.5 ± 7.9 (mean ± SD)		4 clinical variables: PI, BI, CAL, and PPD. The degree of periodontitis was defined by the % of sites with CAL > 3 mm: 0% = absent; 0-32% = slight; = 33-66% = moderate; and 67-100% = severe	Guided anamnesis; general/neurological examination; extensive neuropsychological evaluation; behavioral and functional assessment; complementary examinations (blood tests and structural neuroimaging); DSM-IV for dementia and NINDS-ADRDA for AD	Age, sex, education level, oral hygiene habits, hyperlipidemia, number of teeth	PD (mm) (mean + SD) G1: 3.0 ± 0.7; G2: 2.6 ± 1.5 (p <0.001). CAL (% > 3 mm) (mean + SD); G1: 75.0 ± 28.8; G2: 65.3 ± 35.2 (p = 0.002). In the presence of severe periodontitis: CAL (> 3 mm), n (%); G1: 123 (68.3); G2: 126 (55.0); OR = 3.04, 95% CI: 1.69-5.46, p < 0.001
Ide et al. (2016) [44]	United Kingdom	Prospective cohort	59 with mild to moderate Alzheimer's dementia at baseline. G1 (with periodontitis): 22. G2 (without periodontitis): 37. At the end of the follow-up: G1: 20 and G2: 32	Mean age at baseline (s.e.): 77.7 (8.6) years. G1: 74.9 (2.0). G2: 79.4 (1.3)	6 months	The diagnosis of periodontitis following the established case definitions by the CDC/AAP was based on the assessment of dental plaque, PD, and BoP	NINCDS-ADRDA criteria with ADAS-COG of Alzheimer's disease and sMMSE tests	Age, sex, cognitive scores (ADAS-COG or sMMSE)	Change in Alzheimer’s Disease Assessment Scale ADAS-COG points (s.e.): periodontitis present: 6.1 (1.2); periodontitis absent: 0.9 (1.2); adjusted mean difference (95% CI), p-value: 4.9 (1.2 to 8.6), p = 0.01. Change in the sMMSE points (s.e.): periodontitis present: -2.5 (0.9); periodontitis absent: -0.7 (0.4); adjusted mean difference (95% CI), p-value: -1.8 (-3.6 to 0.04), p = 0.06
Tzeng et al. (2016) [45]	Taiwan	Retrospective cohort	8828. G1 (PD): 2207; G2 (controls): 6621	Age groups: 20-29 (n = 828); 30-39 (n = 1895); 40-49 (n = 2144); 50-59 (n = 1894); 60-69 (n = 1110); ≥70 (n = 957)	10 years	ICD-9-CM codes: 523.4 (chronic periodontitis) and 523.1 (chronic gingivitis)	DSM-IV or DSM-IV Text Revision	Sex, age group, geographic area of ​​residence, urbanization level of residence, monthly income, comorbidities	Development of dementia: G1 had a higher rate of developing dementia at the end of follow-up than G2 (p = 0.042); G1: 25 (1.13%); G2: 61 (0.92%). Development of Alzheimer’s dementia: G1: 4 (16%); G2: 10 (16.4%). At the end of the follow-up: Crude HR = 2.085 (95% CI: 1.297-3.352, p = 0.002). Adjusted HR: 2.54 (95% CI: 1.552-4.156, p < 0.001)
Chen et al. (2017) [46]	Taiwan	Retrospective cohort	G1 (CP): 9291; G2 (controls): 18672	≥50 years. 3 age groups: 50-59 years (n = 13947); 60-69 years (n = 8853); ≥70 years (n = 5163)	G1: 9 to 16 years; G2: not clear	ICD-9-CM diagnostic criteria: code 523.4 (CP)	ICD-9-CM code 331.0	Sex, age group, urbanization level, comorbidities	G1 (AD): 115 (1.24%); G2 (AD): 208 (1.11%). After 10 years of CP exposure: Crude HR = 1.364, 95% CI (1.079-1.725), p = 0.0095. Adjusted HR = 1.707, 95% CI (1.152-2.528), p = 0.0077
Holmer et al. (2018) [47]	Sweden	Case-control	G1 (cases): 154. AD: 52; MCI: 51; SCD: 51. G2 (controls): 76	50-80 years. Median age: G1: 70 years; G2: 67 years. p-value: 0.134		Evaluation of oral hygiene, PPD, BoP, suppuration, tooth mobility, and furcation involvement. Panoramic radiographic imaging: MABL was defined as follows: no/mild (loss of supporting bone <1/3 of root length); local (loss of supporting bone tissue ≥ 1/3 of root length in <30% of the teeth); general (loss of supporting bone ≥ 1/3 the root length in ≥ 30% of teeth)	Dementia: MMSE or MoCA (CDT), blood tests, brain imaging (MRI/CT), EEG, and lumbar puncture with CSF analysis, as well as an extensive neuropsychological assessment. Diagnostic criteria of the 10th revision of ICD and NIA-AA diagnostic guidelines for AD	Age, sex, marital status, education, smoking, body mass index, diabetes mellitus	In the AD subgroup, adjusted OR for generalized MABL: 5.99 (1.02-35.13), p = 0.047. For PPD, the largest association was observed between AD and the presence of one or more pockets ≥ 6 mm, adjusted OR = 15.12 (5.93-38.58), p <0.001

NOS Assessment Results

Assessment results according to NOS are shown in Table 2 for cohort studies and in Table 3 for case-control studies. NOS scores ranged from two to nine points. Three studies had a low risk of bias: one of cohort design [44] and two of case-control design [43,47]. Three other cohort studies were of low quality [42,45,46], while one of the case-control studies accomplished the full score [47].

**Table 2:** Quality assessment results of cohort studies with the Newcastle-Ottawa Scale

Studies	Selection				Comparability	Outcome			Score	Study quality
	Representativeness of the exposed cohort	Selection of the non-exposed cohort	Ascertainment of exposure	Demonstration that outcome of interest was not present at the start of the study	Comparability of cases and controls on the basis of the design or analysis	Assessment of outcome	Was follow-up long enough for outcomes to occur?	Adequacy of follow-up of cohorts		
Arrivé et al. (2012) [42]	0	0	0	1	0	1	0	0	2	Low
Ide et al. (2016) [44]	1	1	1	1	2	1	0	1	8	Good
Tzeng et al. (2016) [45]	0	0	0	1	2	1	1	1	6	Low
Chen et al. (2017) [46]	0	0	0	1	2	0	1	0	4	Low

**Table 3:** Quality assessment results of case-control studies with the Newcastle-Ottawa Scale

Studies	Selection				Comparability	Exposure			Score	Study quality
	Is the case definition adequate?	Representativeness of the cases	Selection of controls	Definition of controls	Comparability of cases and controls on the basis of the design or analysis	Ascertainment of exposure	The same method of ascertainment for cases and controls	Non-response rate		
Gil-Montoya et al. (2015) [43]	1	1	0	1	2	0	1	1	7	Good
Holmer et al. (2018) [47]	1	1	1	1	2	1	1	1	9	Good

Serious limitations were found regarding inconsistency and publication bias in most studies. The exposed cohort of Arrivé et al.'s study was not representative of the population given the lack of information regarding exposure characteristics to PD [42]. In addition, this study was without a control group (unexposed cohort). In the studies of Tzeng et al. and Chen et al. [45,46], cohort selection was considered unrepresentative because samples were selected from a national registry (Taiwan National Health Insurance Research Database), which is a database usually used in descriptive studies. The selection of exposed and unexposed cohorts in Tzeng et al.'s study was affected by the inclusion of young subjects (<40 years old) [45]. Control subjects in Gil-Montoya et al.'s study were from a primary health center rather than the community, thus representing only one particular group and not the general population [43].

Arrivé et al. used the CPI to assess exposure to PD [42]. Although the index includes periodontal pocket depth, it has been considered methodologically deficient as a screening and diagnostic tool [48,49]. In addition, Tzeng et al. and Chen et al. did not mention any description of periodontal assessment [45,46]. The diagnosis criteria for PD in Gil-Montoya et al.'s study were not appropriate since the severity of the disease was defined by the percentage of sites with a clinical attachment loss > 3 mm, while the extent of PDs differs from severity [43]. Regarding AD evaluation, Chen et al. did not report the criteria and tools used to diagnose AD [46].

In terms of follow-up, Ide et al.'s study was of short duration (six months) [44]. Regarding Arrivé et al.'s study, the follow-up from the exposure until the end was not clear [42].

Concerning the adequacy of the follow-up of cohorts, the follow-up in Arrivé et al.'s study was unclear when compared to the number of visits reported by the authors [42]. Furthermore, Chen et al. did not elucidate the loss to follow-up rate in the unexposed cohort [46].

Discussion

Despite the high risk of bias found in most studies included in this systematic review, almost all results demonstrated that patients with PD present an increased risk of developing AD. With the exception of Arrivé et al.'s study, which showed that PD was not associated with the risk of dementia [42], all other studies proved a statistically significant association between PD and the risk of onset and/or progression of AD [43-47]. These findings corroborate the theory that chronic inflammatory diseases such as PD could play an important role in the pathogenesis of AD by progressively inducing neurodegenerative changes leading to disease development.

From an immuno-inflammatory perspective, some authors have hypothesized that there is a positive association between periodontal inflammation and dementia, particularly AD. PD could lead to the development and progression of AD by increasing pro-inflammatory cytokine levels. These pro-inflammatory factors are suggested to be able to invade the blood-brain barrier during inflammatory conditions and to generate neurodegenerative changes and thus the development of dementia [22,25,26,28,36,50,51].

In Kamer et al.'s studies, it has been shown that TNF-α and a high number of antibodies targeting periodontal bacteria are associated with AD [35], and that periodontal inflammation could affect cognitive function [20]. In addition, an association between PD and amyloid Aβ has been demonstrated for the first time in men; the clinical parameters of PD in elders with normal cognitive status were positively associated with an increase of amyloid β accumulation in the brain [50]. In their last review, Kamer et al. explored the hypothesis that PD is causally related to AD by reviewing available evidence using Hill's criteria for causation (consistency of association, strength of association, specificity, temporality, biological gradient, plausibility and analogy, coherence, and experimental evidence) [52]. Although the strength of evidence was not sufficient to confirm causality, they concluded that PD, through its inflammatory and bacterial burdens, could be a biologically plausible risk factor for AD. Laugisch et al. suggested that local production of antibodies in cerebrospinal fluid to *Porphyromonas gingivalis* and other periodontal pathogens may occur in patients with dementia [37]. Nevertheless, no specific association has been found between periodontal infection and the onset of AD. Sparks et al. also demonstrated an increase in antibodies to PD bacteria in subjects for several years prior to cognitive impairment and suggested that PD could potentially contribute to the risk of onset/progression of AD [36]. In this sense, it has been proposed that poor oral health and PD could be considered modifiable risk factors for cognitive impairment and dementia, and early treatment of periodontitis may limit the severity and progression of cognitive lesions [22,24,26]. According to Olsen and Singhrao, oral organisms such as spirochetes, *Chlamydia pneumoniae*, *Helicobacter pylori*, herpes simplex virus type I, and *Candida* species could be involved in causing AD, especially, anaerobic bacteria such as treponemes, *P. gingivalis*, *Fusobacterium* and *Actinomyces*, but also facultative anaerobic *Candida* species [27]. Some reports confirm the existence of a potential infectious invasion of *P. gingivalis* or its components in the brain. In transgenic AD-sensitive mice, *P. gingivalis* was able to enter the brain and cause an inflammatory response [53]. In addition, a positive immunofluorescent reaction of *P. gingivalis* lipopolysaccharide has been found in four out of six brain tissue samples from AD [54]. More recently, a mouse model with *P. gingivalis*-induced experimental periodontitis exhibited memory impairment and a significant increase in amyloid plaque loads, as well as high levels of interleukin-1β and TNF-α in the brain [55]. According to the authors, periodontitis caused by this bacteria could exacerbate the deposition of Aβ in the brain, leading to worsening of cognitive impairments, by a mechanism involving brain inflammation. In their cohort study, Lee et al. found that the risk of developing dementia was significantly higher in elderly participants with periodontitis compared to other patients without periodontitis [56]. They suggested that PD may be a modifiable risk factor for dementia. However, it appears from the value of HR (1.16) that this association is not clinically significant. This study was not included in our systematic review because AD was not explicitly included in the forms of dementia.

Previous systematic reviews and meta-analyses investigating the relation between oral health and dementia were based on tooth loss as the exposure of interest instead of periodontal variables (clinical attachment loss, periodontal probing depth). Shen et al. found that tooth loss is a risk factor that could be positively associated with the increased risk of dementia in older people [57], and Oh et al. revealed that more residual teeth were associated with a lower risk of developing dementia later in life [58]. In the same context, the results of the systematic review by Tonsekar et al. [59] were inconclusive regarding tooth loss and PD as risk factors of dementia or cognitive impairment owing to the divergent results between studies. Furthermore, the systematic review conducted by Wu et al. found inconsistent results on the association between oral health and cognitive decline in the elderly, some showed that oral health measures such as the number of teeth and PD were associated with increased risk of cognitive decline or incident dementia, while no association was found in others [60]. In the systematic review and meta-analysis carried out by Leira et al. for dementia [61], the relative risk of severe periodontitis is higher than for moderate periodontitis. However, the insufficient evidence does not permit to fulfill the criterion temporality for causality between PD and dementia of Alzheimer's type [52]. The significance of the level of severity at a specific moment, might not be as critical as the Alzheimer's patient's exposure to the periodontal microorganisms and their potential entry into the brain over the course of the patient's life, facilitated by different circulatory, lymphatic, neural, and other routes [62]. The review of Leira et al. [61] included all the studies on the association between PD and AD independently of the direction (which disease impacts the other). In the present review, we have focused our review question on the effect of PD on AD. Dioguardi et al. focused on the etiopathogenetic role of PD and periodontal bacteria in the onset and progression of AD compared to unaffected patients [63]. Their systematic review was based on recent literature reviews, in vitro experiments, and clinical studies investigating the role of PD and bacteria such as *P. gingivalis*, *Aggregatibacter actinomycetemcomitans*, *Fusobacterium nucleatum*, and *Treponema denticola* in the onset and progression of AD. They deduced that there is no definitive evidence to consider periodontitis as a risk factor. The systematic review and meta-analysis of observational studies carried out by Nadim et al. to investigate the influence of PD on dementia found that there was an overall significant and positive association of PD with increased dementia with no evidence of the impact of PD on AD [64]. The pooled RR of dementia in relation to PD from all high-quality studies included was 1.38 (95% CI: 1.01-1.90). Nevertheless, the NOS quality assessment of this review reported good quality of three cohort studies [42,45,46], while we evaluated them as having low quality. Furthermore, the authors studied the risk of dementia with their subclassification. In the present review, we opted to focus on AD for more precision.

The present systematic review confirms the findings of Kaliamoorthy et al. [65], despite some differences in eligibility criteria and search strategy leading to the inclusion of other studies [42,45-47]. Recent reviews focusing on the treatment of neuro-inflammation in AD have shown interesting therapeutic results, especially at the early stages of the disease. However, data remain controversial regarding the benefits of non-steroidal anti-inflammatory drugs (NSAIDs) in reducing the risk and progress of AD [66]. Data related to targeting insulin resistance and antibiotic use have been inconclusive as well [67,68].

The restoration and maintenance of healthy gut microbiota could play a key role in the prevention and treatment of AD [67]. It has been proposed that management of the gut microbiota by probiotics may prevent or alleviate the symptoms of these chronic diseases and contribute to reducing neuroinflammation in AD. However, studies that evaluate the potential benefit of probiotic supplements in the course of AD are still needed [67,68].

Nutrition was suggested to be a good measure for the optimization of cognition and prevention of AD’s progress [67,69]. In fact, it was shown that adopting the Mediterranean dietary pattern can play a positive role in cognitive health among healthy individuals and reduce their risk of developing AD [69]. Furthermore, studies included in a recent systematic review have introduced some evidence suggesting a direct relation between diet and changes in brain structure and activity and that higher adherence to a Mediterranean-type diet was associated with decreased cognitive decline [70].

When investigating the relationship between dental treatment and AD, it was shown that periodontal treatment contributed to reducing comorbidities associated with AD [71]. In an analysis that emulates a trial, a moderate to strong effect of periodontal treatment and subsequent maintenance treatment was found on an imaging marker of AD [72]. Hence, PD may be considered a modifiable risk factor for the onset of AD [72,73].

A structured search approach and quality assessment were applied. However, the presence of bias in the studies requires caution while interpreting the results. Several methodological limitations were found while conducting this review. First, there was a large diversity regarding sample size among studies as well as the existence of different follow-up periods. Although the review is based mainly on cohort studies [42,44-46], which have a better level of evidence than cross-sectional and case-control studies, two of the selected studies were case-control [43,47]. This study design prevents the establishment of a causal relationship and cannot infer that PD is a risk factor for AD. For example, in the study by Holmer et al., it is impossible to determine whether the destruction of periodontal tissue occurred before the actual onset of AD [47]. Although PD is a chronic progressive disease and bone loss usually takes many years to occur, the risk of potential reverse causality must be considered, that is, cognitive function leads to poor oral health. In addition, three cohort studies [42,45,46] were of low quality, of which two were retrospective [45,46]. This design has the same level of evidence as case-control studies. Studies that are based on medical records for the diagnosis of dementia have a higher risk of bias than prospective population-based studies, which recruit samples according to adapted evaluation methods and follow-up [74]. Similarly, the lack of consistency in periodontal assessment among studies and in the definition of chronic periodontitis makes it difficult to properly assess the effect of PD on AD. As part of the 2017 global workshop on the classification of periodontal and peri-implant diseases, case definitions for periodontitis, which can be implemented in clinical practice, research, and epidemiological surveillance, have been developed [75]. There was also considerable heterogeneity regarding the criteria and cognitive assessment tools used to diagnose AD and assess the cognitive status of the participants. Another limitation was the incomplete adjustment of confounding factors. An analysis of confounders in observational studies evaluating the association between AD and PD indicated a lack of consideration for potential bias due to confounding [76]. This interferes with the confirmation that PD is a risk factor for AD and its causality or specific role. Although studies have attempted to take these factors into account, residual confusion (due to unmeasured or unknown confounders) is still an impediment to the validity of the results [77]. It has been established that genetics can play a role in the expression of AD. Among the various phenotypes considered, the apolipoprotein E (ApoE) e4 allele is most strongly associated with AD and is considered a major risk factor [78]. In this review, no article evaluated the genotype of ApoE participants as a possible confounding factor in the association between PD and AD. Smoking has been reported in two studies [42,47], whereas no information concerning this variable was given in the other studies. One study included alcohol consumption [42], and three reported a history of depression [42,45,46], while only one took into account oral hygiene habits [43]. In addition, participants' education levels were included in only three studies [42,43,47]. Potential selection bias may exist as participants with a high level of education are more likely to request a dentist's assessment for gingivitis or periodontitis and to seek medical care for a cognitive impairment. In addition, other relevant information regarding other residual confounding factors, such as dietary factors or drug intake, was not included in these studies. These factors may have affected the results reported. It should also be noted that in Arrivé et al.'s study, the statistical calculation of the HR of the periodontal state in association with the AD took as a modality of reference the number of pockets ⩾4 mm instead of healthy periodontal status [42]. This did not make it possible to determine the HR of the modality ⩾4 mm, which remains the most relevant in comparison with the gingival bleeding and the healthy state.

A meta-analysis was not performed because of heterogeneous study protocols and the low number of studies with association measures. For future research, longitudinal studies with a formal diagnosis of AD would be imperative. In addition, standardization of cognitive status assessment would ensure better interpretation of results. These studies must be well-designed and exhaustively define PD (stage and grade). The currently recommended criteria would serve to unify periodontal assessment. They should also include homogeneous and representative study samples and have an adequate follow-up period. The use of standard covariates involved in the interaction between these two diseases as well as a global integration of confounding factors in the statistical analysis would minimize the risk of bias. Randomized clinical trials on the effect of periodontal treatments on the course of AD are also required. Thereafter, more effective methods of patient management could be developed.

## Conclusions

Data from this systematic review indicate that patients with PD present a significantly higher risk of AD compared to periodontally healthy individuals. However, results should be interpreted with caution given the methodological limitations found.

Understanding the contribution of PD to the etiopathogenesis of AD would be of great benefit to the elderly population around the world. For future research, powerful and comparable epidemiological studies are needed to evaluate the effect of PD on cognitive function and AD. Despite the lack of evidence on the effect of PD on AD, it is of vital importance to improve the oral health of subjects with AD and to provide regular dental and periodontal care. A collaboration between dentists and neurologists is also warranted to ensure better care for the patient.
